# Diagnostic accuracy of CBCT versus intraoral imaging for assessment of peri‐implant bone defects

**DOI:** 10.1186/s12880-021-00557-9

**Published:** 2021-02-10

**Authors:** Dandan Song, Sohaib Shujaat, Karla de Faria Vasconcelos, Yan Huang, Constantinus Politis, Ivo Lambrichts, Reinhilde Jacobs

**Affiliations:** 1 OMFS IMPATH Research Group, Department of Imaging and Pathology, Faculty of Medicine, KU Leuven and Oral and Maxillofacial Surgery, University Hospitals Leuven, Kapucijnenvoer 33, 3000 Leuven, Belgium; 2 West China College of Stomatology, State Key Laboratory of Oral Disease & National Clinical Research Center for Oral Disease, Sichuan University, Chengdu, China; 3 Department of Morphology, Biomedical Research Institute, Hasselt University, Diepenbeek, Belgium; 4 Department of Dental Medicine, Karolinska Institute, Stockholm, Sweden

**Keywords:** Alveolar bone loss, Peri‐implantitis, CBCT, Dental radiography

## Abstract

**Background:**

Early detection of marginal bone loss is vital for treatment planning and prognosis of teeth and implant. This study was conducted to assess diagnostic accuracy of CBCT compared to intra-oral (IO) radiography for detection, classification, and measurement of peri-implant bone defects in an animal model.

**Methods:**

Fifty-four mandible blocks with implants were harvested from nine male health adult beagle dogs with acquisition of IO, CBCT and micro-CT images from all samples. Peri-implant bone defects from 16 samples were diagnosed using micro-CT and classified into 3 defect categories: dehiscence (n = 5), infrabony defect (n = 3) and crater-like defect (n = 8). Following training and calibration of the observers, they asked to detect location (mesial, distal, buccal, lingual) and shape of the defect (dehiscence, horizontal defect, vertical defect, carter-like defect) utilizing both IO and CBCT images. Both observers assessed defect depth and width on IO, CBCT and micro-CT images at each side of peri-implant bone defect via CT-analyzer software. Data were analyzed using SPSS software and a *p* value of < 0.05 was considered as statistically significant.

**Results:**

Overall, there was a high diagnostic accuracy for detection of bone defects with CBCT images (sensitivity: 100%/100%), while IO images showed a reduction in accuracy (sensitivity: 69%/63%). Similarly, diagnostic accuracy for defect classification was significantly higher for CBCT, whereas IO images were unable to correctly identify vestibular dehiscence, with incorrect assessment of half of the infrabony defects. For accuracy of measuring defect depth and width, a higher correlation was observed between CBCT and gold standard micro-CT (r = 0.91, 95% CI 0.86–0.94), whereas a lower correlation was seen for IO images (r = 0.82, 95% CI 0.67–0.91).

**Conclusions:**

The diagnostic accuracy and reliability of CBCT was found to be superior to IO imaging for the detection, classification, and measurement of peri-implant bone defects. The application of CBCT adds substantial information related to the peri-implant bone defect diagnosis and decision-making which cannot be achieved with conventional IO imaging.

## Background

Peri-implantitis is a pathological inflammatory reaction leading to progressive loss of the supporting bone which exceeds the physiological bone remodeling around an implant [1, 2]. To date, numerous studies have demonstrated the importance of radiographic imaging modalities for the diagnosis of bone defects [3–5]. An accurate radiographic assessment of the morphology and size of the peri-implantitis bone defect is of vital clinical importance as it directly influences the implant survival and therapeutic outcome of both surgical and non-surgical defect treatment [6–8].

Two-dimensional (2D) imaging modalities such as intraoral (IO) and panoramic radiography are the most commonly utilized radiographic methods for defect detection in a clinical practice based on their low radiation exposure and cost-effectiveness [9–13]. However, they have certain limitations such as, 2D representation of three-dimensional (3D) anatomical structures, geometric distortion, lower spatial resolution, and image magnification which underestimates the defect [14–17]. Furthermore, their inability to diagnose and distinguish buccally and lingually located defects may lead to an inaccurate representation of the bone defect [18, 19]. To overcome the limitations associated with 2D radiography, cone beam computed tomography (CBCT) has been proposed and recommended by various studies as a modality of choice for assessment of periodontal bone defects [20–23]. Undoubtedly, CBCT offers higher accuracy compared to its 2D counterparts for an earlier bone defect detection, thereby allowing immediate application of interventions for controlling further bone loss. Nevertheless, only a few studies are available assessing the superiority of CBCT over 2D imaging for the assessment of peri-implant bone defects [23, 24].

Furthermore, microscopic computed tomography (micro-CT), one of the most versatile non-invasive investigative techniques has been regarded as a standard tool for quantifying the density and architecture of bone in preclinical investigations. Micro-CT functions by illuminating a rotated object with x-rays and collects the magnified projection images via planar x-ray detector. Thereafter, multiple angular images are obtained and stacked together to form the 3D image. Henceforth, micro-CT not only has the ability of imaging the internal biological structures without the need for sample preparation but also provides with 3D representation of the anatomical structures. Previous studies have widely reported on the feasibility and reliability of using micro-CT to evaluate morphologic characteristics of cortical and trabecular bone in both animal and human models [25–28].

Therefore, the current study was conducted to assess the diagnostic accuracy of CBCT compared to IO radiography using the micro-CT as the standard for the detection, classification, and measurement of peri-implant bone defects in an animal model.

## Methods

**Fig. 1:**
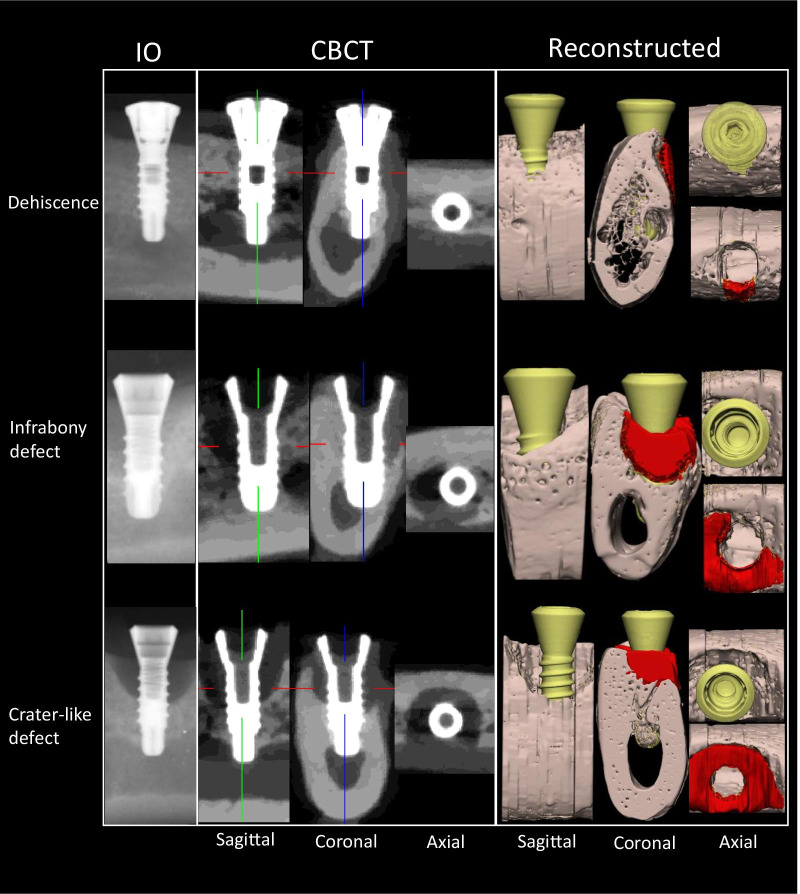
Shapes of peri-implant bone defects which were demonstrated in IO, CBCT and reconstructed imaging

Following ethical approval from the Bioethics Committee of Sichuan University (Reference No: WCCSIRB-D-2014-010), nine health male adult beagle dogs (weight 14–17 kg, age 12–14 months) with completely sound oral condition were recruited by following the ARRIVE guidelines [29] for preclinical animal studies (Supplementary file). All animals were provided by Laboratory Animal Center of Sichuan University. An identical housing and feeding condition was required for all the animals at the Experimental Animal Center of Laboratory of Biotherapy. With injecting general anesthesia with Sumianxin (0.1 ml/kg xylazine hydrochloride, Changchun Military Academy of Medical Sciences, Changchun, China) and local anesthesia (2–4 ml lidocaine 2% epinephrine, Tianjin Pharmaceutical Co. Ltd, Tianjin, China) at the surgical sites, a total of 54 screw-type titanium dental implant with plasma-sprayed hydroxyapatite (HA) coating (3.3 mm Ø × 8 mm, cylindrical, non-submerged healing, BLB, China) were inserted in the mandibular region of each dog (n = 6 per dog). The sample size was calculated based on a prior power analysis in G*power 3.1 at a power of 80% [30]. Following crown preparation and attachment, each implant was followed-up for a period of at least 1 months. The surgical procedure has been explained in a prior publication [31, 32]. Thereafter, all animals were euthanized using an overdose of xylazine hydrochloride (intravenous injection) and immediate perfusion of 4% paraformaldehyde and 0.0125% glutaraldehyde in 0.1 M phosphate buffer (pH 7.4). All dogs were healthy with clinically stable implants before sacrifice. The mandible blocks with implants were harvested and IO, CBCT and micro-CT images were acquired for each sample, where micro-CT acted as the gold standard. Table 1 describes the details of the acquisition devices and scanning parameters. Following image acquisition, 16 samples were found to have bone defects and were included in the study. The marginal bone level around the implant lower than the first screw loop of the implant from the top was judged as the bone defect; otherwise, the others were excluded. All the images were manually reoriented along implants long axis with DataViewer (ver. 1.5.1.2, Bruker). Following orientation, three types of bone defects were recognized and diagnosed using micro-CT, which included dehiscence (n = 5), infrabony defect (n = 3) and crater-like defect (n = 8) (Fig. 1). The diagnosis was carried out by a consultant oral and maxillofacial radiologist with an experience of over 20 years. Later, two dentists were recruited as observers with an experience of at least 5 years in dental imaging. Following training and calibration of the observers, all the samples marked by the implant site were renumbered and randomized by the method of random sort in Excel. The observers were asked to detect the location of the defect (mesial, distal, buccal, lingual) and the shape of the defect (dehiscence, horizontal defect, vertical defect, crater-like defect). All evaluations were performed with both IO and CBCT images.

**Table 1:** Details of the protocols for each acquisition device and scanning parameter

	Intraoral radiography	Cone-beam computed tomography	Micro-CT
Product name	Heliodent Plus	3D Accuitomo 170®	Quantum FX
Company	Sirona Dental Systems GmbH Bensheim, Germany	J. Morita Kyoto, Japan	PerkinElmer, Inc. Waltham, USA
Tube current (mA)	7	5	0.16
Tube voltage (kV)	60	90	90
Voxel size (mm)	–	0.08	0.02
Field of view (cm)	3.3 × 4.3	10 × 5	0.01 × 0.01
Exposure time (s)	0.12	17.5	180

**Fig. 2:**
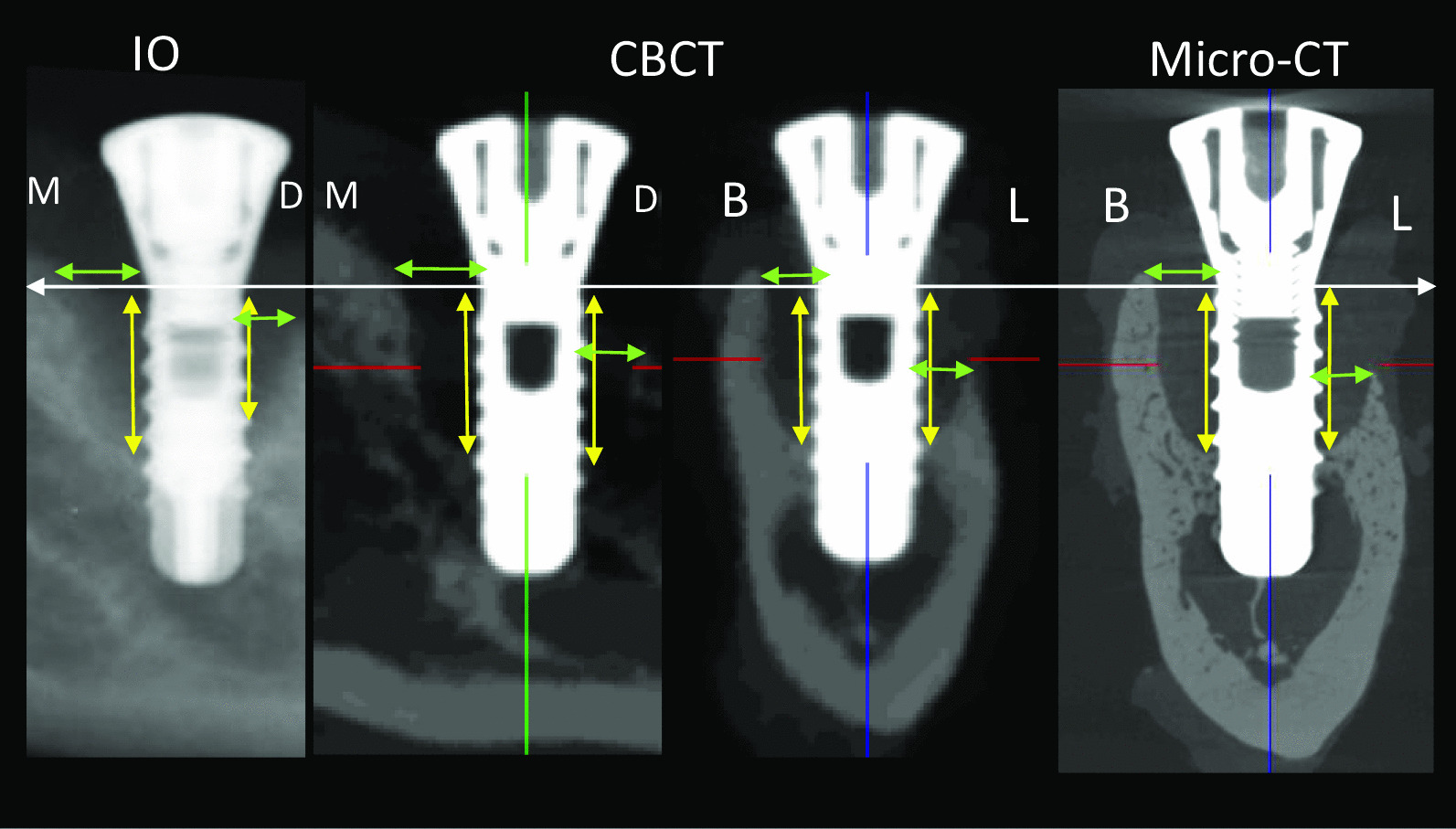
Method of depth and width measurement in intra-oral radiography (IO), cone-beam CT (CBCT) and micro-CT imaging. White arrow, implant shoulder as reference; Yellow arrow, depth of bone defect (from implant shoulder to the most apical of bone defect); Green arrow, width of bone defect (from implant shoulder to bone crest)

Following diagnosis, both observers measured the defect depth and width on IO, CBCT and micro-CT images at each side of peri-implant bone defect via CT-analyzer software (version 1.16.1.0, Skyscan1272, Bruker MicroCT, Kontich, Belgium). The slices in 3D images were standardized to that of the 2D image. A mesio-distal and bucco-lingual slice was selected from the sagittal and coronal view individually on the 3D images and were oriented parallel to the long axis of implant. The width and depth of the defects were measured as shown in Fig. 2. The observers performed both diagnosis and measurement tasks at a two weak interval with randomization of the data for assessing the observer reliability.

### Statistical analysis

Data were analyzed using SPSS software (Version 22, IBM, New York, USA). The diagnostic accuracy of IO and CBCT images for the defect detection and classification was assessed by calculating the sensitivity and intra- and inter‐rater reliability (Cohen’s and Fleiss’s Kappa) of each method and observer. The interpretation of Kappa values was carried out as suggested by Landis and Koch [33]. Intra-class correlation coefficient (ICC) was utilized for calculating the absolute inter-protocol, intra-rater, and inter-rater agreement for defects depth and width measurements. Kruskal-Wallis one-way ANOVA test was performed to compare the depth and width of the infrabony defect with that of the crater-like defect. A *p* value of < 0.05 was considered as statistically significant.

## Results

Overall, a high agreement (Kappa: 0.92; 95% CI 0.88–0.96) and reliability (ICC: 0.97; 95% CI 0.96–0.97) was observed for the detection and classification of the defect on IO and CBCT images. Intra- and inter-rater agreement for the diagnosis with CBCT was found to be almost perfect for each observer. The reliability of CBCT images was also considerably high when compared with the micro-CT for the detection and classification of the bone defect (ICC: 0.93 and 0.96, 95% CI 0.9–0.95 & 0.95–0.98). Additionally, the intra-rater and inter-rater agreement between both observers was higher with CBCT compared to IO images (Table 2). Both observers showed a high diagnostic accuracy for detection of the bone defect with CBCT images (sensitivity: 100%/100%), whereas, IO images showed a reduction in the accuracy (sensitivity: 69%/63%). Similarly, the diagnostic accuracy for the classification of the defect was also higher for CBCT when compared with IO images (Table 3).

**Table 2:** Reliability and agreement in detection of the bone lesion and morphology classification

Detection parameters	Methods	Observer effect			
		Reliability		Agreement	
		ICC	95% CI	Weighted Kappa	95% CI
Bone defect presence	IO	0.96	0.94–0.97	0.84	0.72–0.96
	CBCT	0.97	0.96–0.98	0.87	0.74–0.99
	Total	0.90	0.86–0.92	0.78	0.68–0.88
Shape classification	IO	0.96	0.94–0.97	0.84	0.72–0.94
	CBCT	1	–	0.94	0.97-1
	Total	0.92	0.90–0.94	0.82	0.74–0.90
Total		0.97	0.96–0.97	0.92	0.88–0.96

Reliability, intra-class correlation coefficient; Agreement, inter-rater agreement; IO, intra-oral radiography; CBCT, cone beam computer tomography

**Table 3:** Sensitivity of the diagnosis of bone defect presence and shape

Observers	Methods	Bone defect presence (%)	Bone defect shape		
			Dehiscence	Infrabony defect (%)	Crater-like defect (%)
1	IO	69	0	50	78
	CBCT	100	100%	100	90
2	IO	63	0	50	78
	CBCT	100	100%	100	89

IO, intra-oral radiography, CBCT, cone beam computer tomography

For the accuracy of measuring depth and width of the defect, the ICC and agreement with the gold-standard micro-CT was found to be approximately 1 (ICC: 0.99, 95% CI 0.996–0.998; Kappa: 0.91). A higher correlation was observed when CBCT was compared with micro-CT (r = 0.91, 95% CI 0.86–0.94), whereas slightly lower correlation was seen with IO images (r = 0.82, 95% CI 0.67–0.91).

**Fig. 3:**
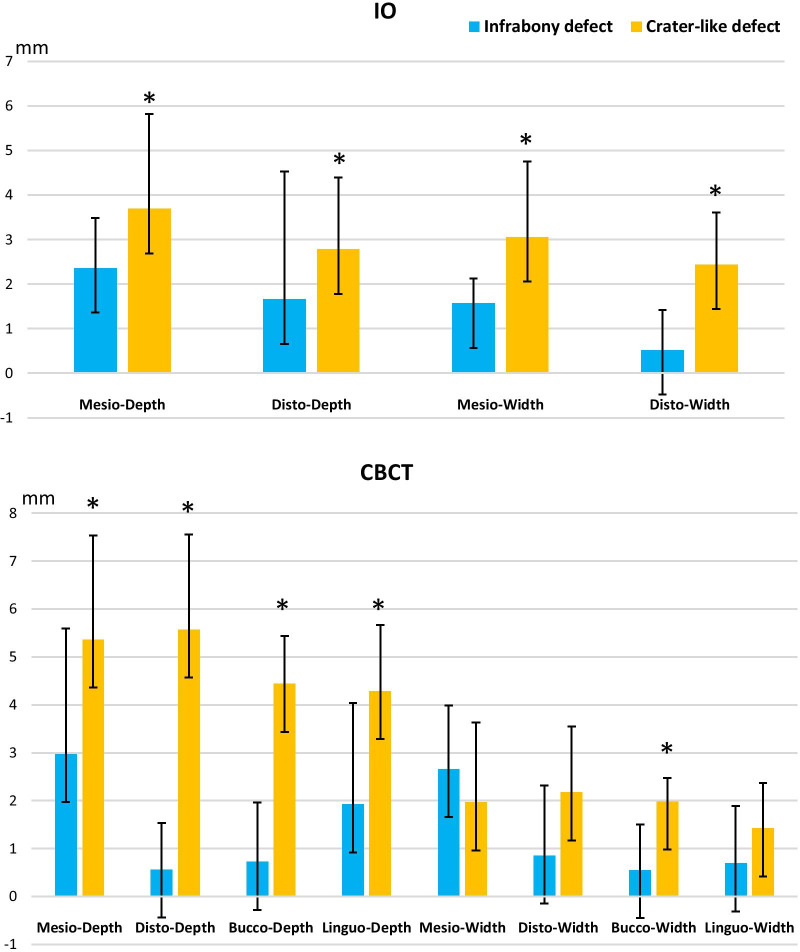
Graph of bone defect size (depth and width) for different shapes of peri-implant bone lesion in IO and CBCT imaging. *p < 0.05. IO: Intra-oral radiography; CBCT: cone beam computer tomography

The relationship between the bone defect morphology and size is demonstrated in Fig. 3. The depth for crater-like defect was larger than infrabony defect at all sides in both IO and CBCT images. The distal (*p* = 0.003) and buccal side (*p* = 0.002) showed a significantly larger depth in crater-like defects. Similarly, a larger defect width was observed for crater-like defects in both IO and CBCT images, except mesial side on CBCT images which showed a larger width in infrabony defect. Whereas, IO images showed a significantly larger width on the mesial side of the crater-like defect.

## Discussion

Evidence suggests multiple studies underlining the importance of the accurate detection and classification of bone defect which can influence the treatment planning and prognosis of dental implant [34–36]. Therefore, the present study was conducted to assess the diagnostic accuracy of CBCT and IO imaging for the detection, classification, and measurement of peri-implant bone defects.

Our findings suggested a higher reliability and diagnostic accuracy for the detection and classification of peri-implant defects with CBCT compared to IO images. Based on the 2D nature of IO radiographs, it only allowed accurate diagnosis of the horizontal and 1-wall vertical bone defects in a mesiodistal direction [5]. This could have led to a higher sensitivity of CBCT which offers 3D visualization of the bone. These findings were consistent with previous studies, which suggested CBCT to have a significantly higher diagnostic accuracy compared to digital periapical radiography for the detection of fenestration, dehiscence and three-walled periodontal defects [23, 24, 37]. In a study by Noujeim, et al. [22], a high sensitivity was recorded for the detection of large bone defects by both IO and CBCT imaging and they suggested CBCT imaging only for small bone defects of < 1mm where IO radiography was considered insufficient for an accurate diagnosis. However, their study has limited clinical value as they observed defects only in the mesiodistal direction, and buccolingual aspect of the defect was not included in the evaluation. Whereas, in our study five bone defects were with dehiscence on the lingual side which could not be detected or classified accurately on IO radiographs, thereby leading to a further reduction in its diagnostic accuracy [5]. Similarly, inconsistency was observed with a study by Kühl, et al. [16]. where authors found that IO radiography offered improved accuracy compared to CT-based images for the detection of peri-implant bone defect. The main reason for decreased accuracy of CT-based images in their study was based on the presence metal artefacts resulting in lower quality images. On the other hand, we acquired images with a high-resolution CBCT protocol and the CBCT device had an inbuilt metal artefact reduction algorithm resulting in high quality images.

In relation to the mesiodistal depth and width measurements of the bone defect, a high correlation was found for both IO (r = 0.82) and CBCT (r = 0.92) images when compared with the micro-CT. The slightly lower correlation value with IO could have resulted due to the overlapping effect and blurring of the peri-implant region. We found the size of defect to be significantly larger in crater-like defect compared to infrabony defect. A previous study observed peri-implant bone defects to be larger on buccal side compared to other walls in patients with peri-implantitis [38]. In contrast, we found defects to be larger mesiodistally followed by lingual and buccal wall. The difference in study design where we utilized an animal model and location of the implant placement could have resulted in these inconsistent findings. The detection, classification and accurate measurements of the defect are all critical parameters as they might influence the success of an implant or regenerative therapy if required [39]. Although CBCT carries the risk of higher radiation dose compared to IO radiography, it can be argued whether the benefit of an accurate diagnosis with CBCT outweighs the risks involved with a higher radiation dose. We believe that a clinician should not hesitate to order a CBCT for the diagnosis and follow-up of a peri-implant defect, as IO radiography is insufficient for an accurate diagnosis [40]. At the same instance, the scanning parameters should be optimized accordingly for reducing the radiation exposure to the patient.

Our study had certain limitations. Firstly, the sample size was not sufficient for drawing a meaningful conclusion related to the relationship between the bone defect type and size. Secondly, the setup for the high resolution CBCT was obviously diverting from that of a normal patient acquisition. We realise that this may cause some deviation when transferring the present imaging data and outcome to the clinic situation. However, the high-dose allowed accurate investigation of infra-bony defects which might get impeded due to the presence of implant-related artefacts if a lower dose is applied. Further studies are required to assess the diagnostic accuracy of low-dose CBCT protocols in patients for detecting and classifying peri-implant bone defects. In the midst of these limitations, we provided evidence related to the detection of bone defects which might not be visible on IO radiographs, thereby leading to an inaccurate diagnosis and treatment planning. Although IO radiography is considered as a clinical standard for assessing peri-implant bone defects [41], the importance of CBCT imaging for diagnosis and follow-up should not be ignored for employing timely management of the defect [42, 43]. Further studies should be carried out with patient specific low-dose CBCT protocols to assess their accuracy for peri-implant bone defect diagnosis. Also, future studies should pay attention to the implant blooming artefacts that are impacting peri-implant visibility and are influenced by CBCT device and protocol characteristics as well as by implant material and design [44, 45].

## Conclusions

The diagnostic accuracy and reliability of CBCT was found to be superior to IO imaging for the detection, classification, and measurement of peri-implant bone defects. The application of CBCT adds substantial information related to the peri-implant bone defect diagnosis and decision-making which cannot be achieved with conventional IO imaging. However, benefit-risk ratio should be kept in mind and CBCT should be acquired for cases where a peri-implant bone defect might influence the implant survival rate.

## Data Availability

The datasets used and/or analyzed during the current study are available from the corresponding author on reasonable request.

## Abbreviations

CBCT: Cone beam computed tomography
Micro-CT: Microscopic computed tomography
IO: Intraoral radiography
2D: Two-dimensional
3D: Three-dimensional
HA: Plasma-sprayed hydroxyapatite
ICC: Intra-class correlation coefficient
